# Effects of Alhagi Honey Polysaccharides as Feed Supplement on Intestine Function and Microbiome, Immune Function, and Growth Performance in Chicken

**DOI:** 10.3390/ijms232214332

**Published:** 2022-11-18

**Authors:** Gaofeng Cai, Ningning Mao, Pengfei Gu, Tianyu Zhu, Jin He, Song Peng, Yang Yang, Zhenguang Liu, Yuanliang Hu, Deyun Wang

**Affiliations:** 1 Institute of Traditional Chinese Veterinary Medicine, College of Veterinary Medicine, Nanjing Agricultural University, Nanjing 210016, China; 2 MOE Joint International Research Laboratory of Animal Health and Food Safety, College of Veterinary Medicine, Nanjing Agricultural University, Nanjing 210095, China

**Keywords:** growth performance, Alhagi honey, polysaccharides, gut microbiome, immune function, poultry

## Abstract

Hy-Line Brown chickens’ health is closely related to poultry productivity and it is mainly maintained by the immune system, healthy intestinal function, and microflora of chicken. Polysaccharides are biological macromolecules with a variety of activities that can be used as a potential prebiotic to improve poultry health. In this experiment, the function of Alhagi honey polysaccharides (AH) as an immunomodulator on the chicken was investigated. All chicken (120) were randomly distributed to four groups (five replicas/group, six hens/replica). A total of 0.5 mL water was taken orally by the chicken in control group. AH (0.5 mL) in different concentrations (three dosages, 0.3 g/kg, 0.6 g/k, and 1.2 g/kg) were used for the AH-0.3 g/kg, AH-0.6 g/k, and AH-1.2 g/kg group, respectively. The results showed that the growth performance of the chickens and the index of immune organs (the weight of immune organs/the body weight) were enhanced significantly after being AH-treated (*p* < 0.05). The content of sIgA and cytokines was upregulated remarkably in the intestine after being AH-treated (*p* < 0.05). The AH treatment significantly enhanced the intestinal epithelial barrier (*p* < 0.05). Moreover, the percentage of CD4^+^ and CD8^+^ T cells in the ileum, spleen, and serum were obviously upscaled (*p* < 0.05). In addition, the AH treatment significantly enhanced the production of short chain fatty acids (SCFAs) and improved the structure of gut microbiota (*p* < 0.05). In conclusion, we found that AH-1.2g/kg was the best dosage to improve the chicken’s health, and these data demonstrated that AH could be used as a potential tool to enhance growth performance through improving intestine function, immunity, and gut microbiome in chicken.

## 1. Introduction

The immune system, as a vital defense system to protect the Hy-Line Brown chickens’ health, consists of a variety of immune organs and immune cells, such as the spleen, thymus, Bursa of Fabricius, intestine, lymphocytes, etc. In particular, the intestine is not only the biggest immune organ in the chicken, but also the largest organ of digestion and absorption [1]. Thus, the health of the intestine was closely related to the growth performance of chicken. The mucosal immune system and intestinal barriers are well known as the important elements in resistance to pathogen invasion and maintaining intestinal health [1,2]. The intestinal immune system, as an independent immune system, has unique structural and functional characteristics and always directly eliminates the foreign antigens through activating T cells and releasing sIgA and cytokines [1,3]. Tight junctions’ (TJs) proteins, goblet cells, intestinal epithelial cells, and mucus proteins were the main tool of the epithelial and chemical barriers in fighting against infections [4]. The biological barrier is composed of a number of microscopic organisms, which are closely correlated with immunity, nutrient digestion, and absorption [5]. If the immune function and intestinal function of chicken are not perfect, they will weaken the disease resistance and the anti-stress ability will be weak, which brings huge health risks to the survival of poultry. Moreover, the intestinal microflora of chicken is easily disturbed by external stress, which may cause enteritis and diarrhea [5]. Therefore, the development of high-efficiency, healthy, and immune-boosting dietary additives to improve the immunity, intestinal function, and gut microbiome is of great significance to the poultry breeding industry.

In recent years, polysaccharides, as an important plant extract compound, have been well known for their anti-inflammatory, anti-cancer, immunomodulatory and microbiome regulating activities [1,2]. Moreover, due to their prominent pharmacological activities, some polysaccharides extracted from plants have been used to improve intestinal health, immunity, and growth performance in intensive farming [6,7]. Furthermore, recent studies have demonstrated that polysaccharides can regulate the intestinal microbiota structure and be fermented by the gut flora to produce the short chain fatty acids (SCFAs), which are further used to alleviate intestinal dysfunction [2,8]. Therefore, polysaccharides may serve as a useful natural dietary additive to improve the immunity, intestinal function, and structure of the intestinal flora of poultry at the chick stage.

Alhagi honey polysaccharide (AH) is natural polymer extracted from Alhagi honey. It has been used in the treatment of diarrhea and applied to improve immune function in the Xinjiang province of China [9]. Additionally, many previous studies have demonstrated that Alhagi honey polysaccharides display various pharmacological activities, including the enhancement of immunity, anti-inflammatory, and anti-bacterial activities, etc. [9,10]. Li Gairu et al. found that AH could enhance the immunity of RAW264.7 macrophages in vitro [11]. Kuerbanjiang Maimaitimin et al. found that alcoholic liver injury could be ameliorated by the AH [12]. Our previous study discovered that a polysaccharide primary, consisting of glucose, galactose, mannose, and rhamnose, with a molecular weight of 46.8 kDa, was extracted from the Alhagi honey [9]. Adelijiang Wusiman determined that AH could be used as an immunologic adjuvant to improve the immunity of mice [9]. In addition, we demonstrated that AH can enhance the intestinal immunity of mice and improve the structure of the flora [3]. However, the effect of AH on chickens’ intestinal function, intestinal microbiota, and immune function are unknown.

Thus, this research will assess the potential of AH as a natural dietary additive to improve chickens’ health by focusing on its effects on immunity, intestinal function, the gut microbiome, and growth performance. We assessed the influence of AH on chickens’ growth performance by recording the body weight change and feed consumption. In addition, the effects of AH on chickens’ intestinal function were analyzed by determining the intestinal morphology, intestinal physiology, immune function, the concentrations of SCFAs, and the intestinal flora structure. Furthermore, the immune organ index and the proportion of CD8^+^ and CD4^+^ T cells in the spleen and peripheral blood were investigated as indices for immune function after AH intake.

## 2. Results

### 2.1. Influence of Alhagi Honey Polysaccharides on the Growth Performance and the Index of Immune Organs

**Figure 1:**
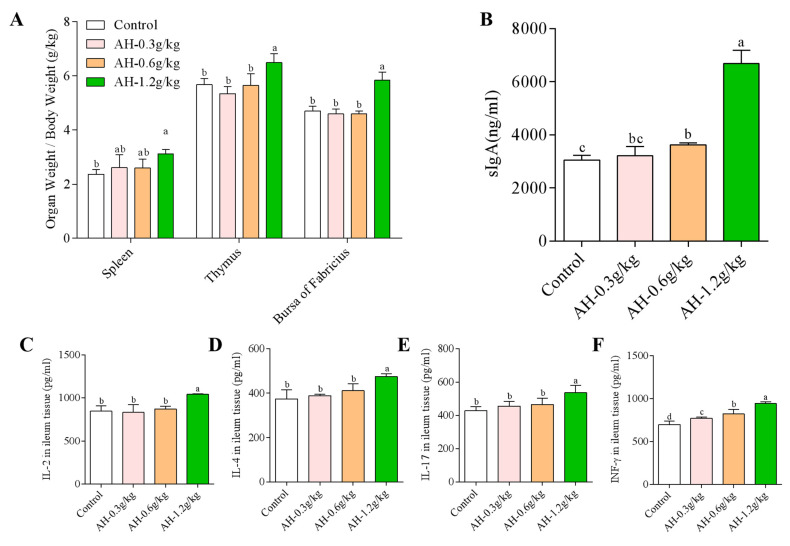

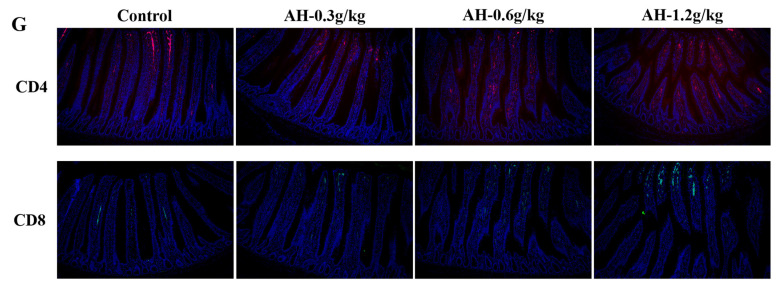
(**A**) Effects of AH on immune organs indices in chicken after the experiment. (**B**) The effect of AH on the secretion of sIgA in the small intestine. The expression level of IL-2 (**C**), IL-4 (**D**), IL-17 (**E**), and IFN-γ (**F**) in duodenum was determined by ELISA. The number of CD4^+^ and CD8^+^ T cell in ileum was measured by immunofluorescence (**G**). CD4^+^ T cells were labeled with CY3 (red points), CD8^+^ T cells were labeled with FITC (green points), and nuclei were labeled with DAPI (blue points). Bars = 100 μm, 100×. Data are given as mean ± SEM. (n = 5). Bars in the figure without the same superscripts (a–d) differ significantly (*p* < 0.05).

In this study, the result of average daily gain (ADG), average daily feed intake (ADFI), and feed-to-gain ratio (F:G) was showed in Table 1. During the AH-treatment (D1-D7) period, the result of ADG, ADFI, and F:G was exhibited no statistical significance among the four groups (*p* = 0.871, *p* = 0.083*, p =* 0.995). However, compared to the control group, the ADG in AH-1.2 g/kg group was increased significantly during the AH-treatment (D8-D14) period (*p* = 0.024). In contrary, compared to the control group, AH-1.2 g/kg group could remarkably decrease the F:G (*p* = 0.011). Additionally, the immune function of thymus, spleen, and BF was observed by detecting the index of thymus, spleen, and BF. The result showed that compared to the AH-1.2 g/kg group, the index of thymus, spleen, and BF in the control group was obviously lower (*p =* 0.015*, p* = 0.016, *p =* 0.001, Figure 1A).

### 2.2. Influence of Alhagi Honey Polysaccharides on the Secretion Level of sIgA in Duodenal

To study the influence of AH on sIgA, we detected the secretion content of sIgA in duodenal after the experiment. As shown in Figure 1B, compared to the control group, the secretion level of sIgA in AH-1.2 g/kg and AH-0.6 g/kg groups was enhanced prominently (*p* = 0.001, *p =* 0.026). Compared to the AH-0.6 g/kg group, the secretion level of sIgA in AH-1.2 g/kg was enhanced significantly (*p* = 0.001).

### 2.3. Influence of Alhagi Honey Polysaccharides on the Secretion Level of Cytokines in Duodenum and the Ratio of CD8^+^ and CD4^+^ T Cells in Ileum

As shown in Figure 1C–G, the secretion content of IL-2, IL-4, IL-17, and INF-γ in AH-1.2 g/kg group was remarkably higher than that in control (*p* = 0.001, *p* = 0.001, *p* = 0.001, *p* = 0.001), AH-0.6 g/kg group (*p* = 0.001, *p* = 0.001, *p* = 0.007, *p* = 0.001), and AH-0.3 g/kg group (*p* = 0.001, *p* = 0.006, *p* = 0.015, *p* = 0.001). Moreover, the secretion level of INF-γ in AH-0.3 g/kg and AH-0.6 g/kg groups was significantly upregulated when compared to the control group (*p* = 0.012, *p* = 0.001). AH-0.6 g/kg and AH-1.2 g/kg groups could remarkably upregulate the ratio of CD8^+^ and CD4^+^ T cells as compared to control group, which was observed by immunofluorescence.

### 2.4. Influence of Alhagi Honey Polysaccharides on Intestinal Morphology and the Number of IELs

**Figure 2:**
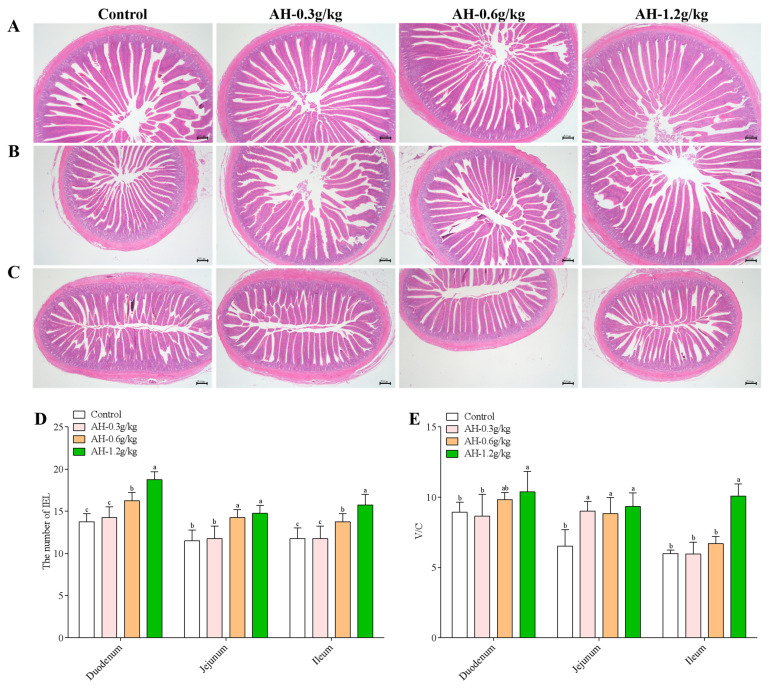
Effects of AH on intestinal mucosal morphology was determined by Hematoxylin-eosin staining (HE) after the experiment. (**A**) duodenum (HE), (**B**) jejunum (HE), (**C**) ileum (HE). (**D**) The number of IELs in duodenum, jejunum, and ileum. (**E**) The ratio of villus height/crypt depth in different sections of intestine. Bars = 100 μm, original magnification, 100×. Data were given as mean ± SEM. (n = 5). Bars in the figure without the same superscripts (a–c) differ significantly (*p* < 0.05).

As shown in Figure 2A–C, the intestinal morphology was no abnormality, the intestinal villi were neatly arranged, and there were no lesions in four groups. In addition, the result of intraepithelial lymphocytes (IELs) was showed in Figure 2D, in the duodenum, jejunum and ileum, the content of IELs could be significantly enhanced by AH-1.2 g/kg (*p* = 0.001, *p* = 0.002, *p* = 0.001) and AH-0.6 g/kg group (*p* = 0.005, *p* = 0.007, *p* = 0.044) as compared with the control group. The content of IELs in the duodenum, jejunum and ileum of AH-0.3 g/kg was significantly lower than that in the AH-1.2 g/kg (*p* = 0.001, *p* = 0.004, *p* = 0.001) and AH-0.6 g/kg group (*p* = 0.019, *p* = 0.012, *p* = 0.044). In duodenum and ileum, the number of IEL in AH-1.2 g/kg group was remarkable higher than that in AH-0.6 g/kg group (*p* = 0.001, *p* = 0.044).

Additionally, as presented in Figure 2E. The ratio of V/C in control and AH-0.3 g/kg group in duodenum was prominently lower than that in AH-1.2 g/kg group (*p* = 0.043, *p* = 0.031). Moreover, in jejunum, AH-treated groups (0.3 g/kg, 0.6 g/kg, 1.2 g/kg) could remarkably enhance the ratio of V/C as compared to control group (*p* = 0.001, *p* = 0.002, *p* = 0.001). In ileum, compared to control (*p* = 0.001) and other two AH-treatment groups (*p* = 0.001, *p* = 0.001), the ratio of V/C in AH-1.2 g/kg group was enhanced prominently.

### 2.5. Influence of Alhagi Honey Polysaccharides on the Number of Goblet Cells, the mRNA Expression of Claudin-1, Occludin, and ZO-1 proteins in Ileum, and the SCFAs Production in Cecum

**Figure 3:**
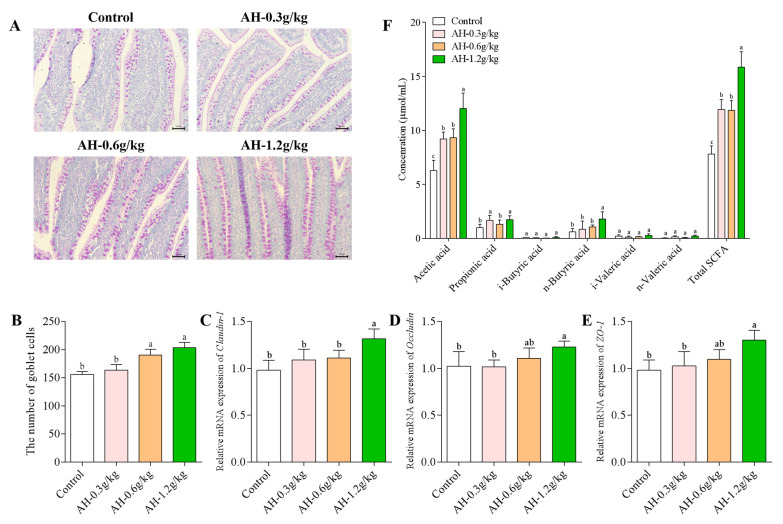
(**A**) The goblet cell was observed by AB-PAS staining in ileum after the experiment. Bars = 200 μm, 50×. (**B**) The number of goblet cells in intestinal mucosal surface were detected. Relative mRNA expressions of Claudin-1 (**C**), Occludin (**D**), and ZO-1 (**E**) proteins in ileum. (**F**) Effects of AH on the SCFAs in the contents of cecum. The expression levels were compared to that of GAPDH which was used as the internal housekeeping gene. Data analysis was carried out using the 2^−ΔΔCT^ method. Data were given as mean ± SEM. (n = 5). Bars in the figure without the same superscripts (a–c) differ significantly (*p* < 0.05).

The number of goblet cells in ileum after AH-treated was presented in Figure 3A,B. Compared to AH-0.6 g/kg and AH-1.2 g/kg groups, the number of goblet cells in AH-0.3 g/kg (*p* = 0.005, *p* = 0.001) and control (*p* = 0.001, *p* = 0.001) groups was remarkable lower. Moreover, as shown in Figure 3C–E. The AH-1.2 g/kg group could significantly enhance the expression of Claudin-1, Occludin, and ZO-1 genes when compared to control (*p* = 0.004, *p* = 0.049, *p* = 0.011) and AH-0.3 g/kg group (*p* = 0.026, *p* = 0.044, *p* = 0.021). Moreover, AH-1.2 g/kg group could remarkably un-regulate the expression of Claudin-1 gene as compared to the AH-0.6 g/kg group (*p* = 0.038).

Herein, the content of microbial metabolites SCFAs was investigated after AH-treated. As presented in Figure 3F, compared to the control group, AH-treated groups (0.3 g/kg, 0.6 g/kg, 1.2 g/kg) could significantly enhance the content of acetic acid (*p* = 0.001, *p* = 0.001, *p* = 0.001) and total acid (*p* = 0.001, *p* = 0.001, *p* = 0.001), and the content of acetic acid (*p* = 0.001, *p* = 0.001) and total acid (*p* = 0.001, *p* = 0.001) in AH-1.2 g/kg group was observably higher than that in other two AH-treatment groups (0.3 g/kg, 0.6 g/kg). Moreover, compared to control and AH-0.6 g/kg groups, the concentrations of propionic acid in AH-1.2 g/kg (*p* = 0.009, *p* = 0.031) and AH-0.3 g/kg (*p* = 0.015, *p* = 0.041) groups were enhanced significantly. AH-1.2 g/kg could significantly increase the concentrations of n-butyric acid as compared to control (*p* = 0.003) and other two AH-treatment groups (*p* = 0.015, *p* = 0.045).

### 2.6. Influence of Alhagi Honey Polysaccharides on the Gut Microbiome

**Figure 4:**
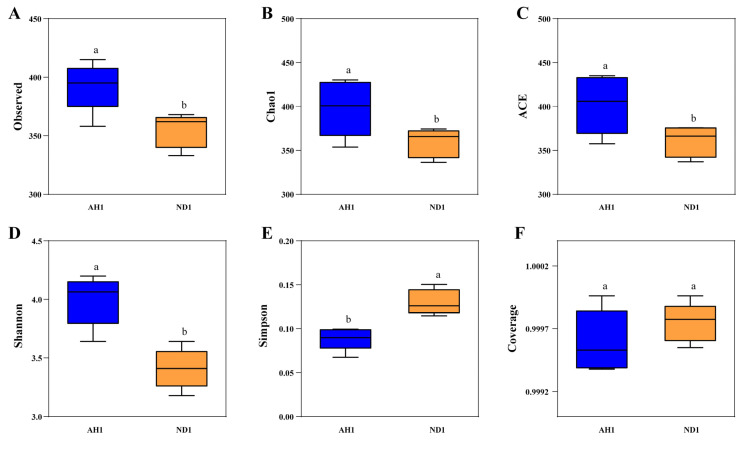

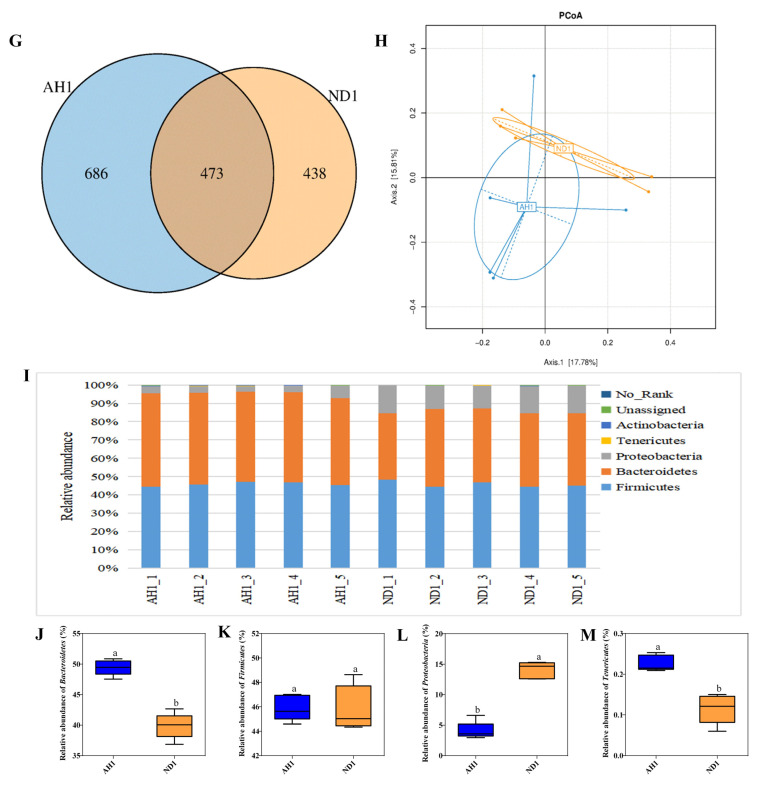
α-diversity and β-diversity analysis of gut microbiome (n = 5 per group). (**A**–**F**) Evaluation of observed richness, diversity and coverage for the different fecal samples. (**G**) Venn diagram based on ASV level. (**H**) Principal Coordinate Analysis (PCoA) of bacterial communities using Jaccard distance. Each point represents a sample, with a shorter distance between the points indicating a greater similarity in microbial community composition between samples. (**I**) Relative abundances (%) of the major bacterial phyla of sample. (**J**) Relative abundance of Bacteroidetes across each grouped microbiome. (**K**) Relative abundance of Firmicutes across each grouped microbiome. (**L**) relative abundance of Proteobacteria across each grouped microbiome. (**M**) Relative abundance of Tenericutes across each grouped microbiome. Data are given as mean ± SEM. (n = 5). Bars in the figure without the same superscripts (a–b) differ significantly (*p* < 0.05).

An analysis of poultry intestinal flora alpha diversity was performed, the result was illustrated in Figure 4A–F. Compared to control group, AH-1.2 g/kg group significantly increase the ASVs number (*p* = 0.011), and the Goods’ coverage of each sample in two groups was higher than 99.9%. The species richness was represented by Chao1 and ACE indices, and Shannon and Simpson indices was used to estimate species diversity. The higher Shannon index suggests the higher the community diversity, but the higher Simpson index suggests the lower the community diversity. As listed in Figure 4B–E, the Chao1 and ACE indices was enhanced remarkably in the AH-1.2 g/kg group as compared to control group (*p* = 0.04, *p* = 0.037). Moreover, compared to AH-1.2 g/kg group, the Shannon indices in control group was obviously lower (*p* = 0.001), but the completely opposite trend was shown on the Simpson indices (*p* = 0.001).

In addition, Beta diversity analysis was shown in Figure 4G–H. The result showed that 686 unique ASVs was in AH-1.2 g/kg group, 438 unique ASVs was in control group, and 473 ASVs were shared by the two groups, but also had differences. Moreover, the principal coordinate analysis (PCoA) showed that obviously separated clustering of bacterial communities was displayed between AH-1.2 g/kg group and control group.

### 2.7. Influence of Alhagi Honey Polysaccharides on Intestinal Flora at the Phylum

In order to better display the regulatory effect of AH on the intestinal flora, we analyzed the gut flora structure at phylum level. The proportion of intestinal microbes was showed in Figure 4I. Firmicutes, Bacteroidetes, Proteobacteria, and Tenericutes were the main cecum bacteria, which made up more than 90% of the relative abundance. In Figure 4J–M, the abundances of Bacteroidetes and Tenericutes in AH-1.2 g/kg group were significantly higher than that in control group (*p* = 0.001, *p* = 0.001). Moreover, compared to control group, AH-1.2 g/kg group could remarkably decrease the abundances of Proteobacteria (*p* = 0.001). Furthermore, AH-1.2 g/kg group could not change the abundances of Firmicutes as compared to control group.

### 2.8. Influence of Alhagi Honey Polysaccharides on Activation of CD4^+^ and CD8^+^ T Cells in Spleen and Peripheral Blood

**Figure 5:**
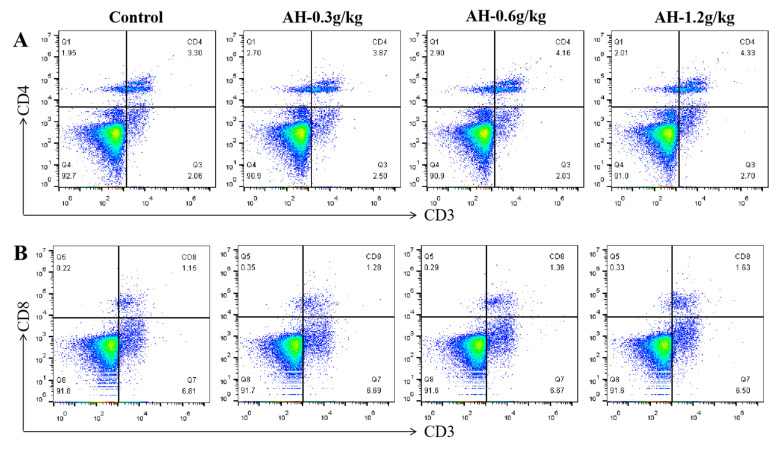

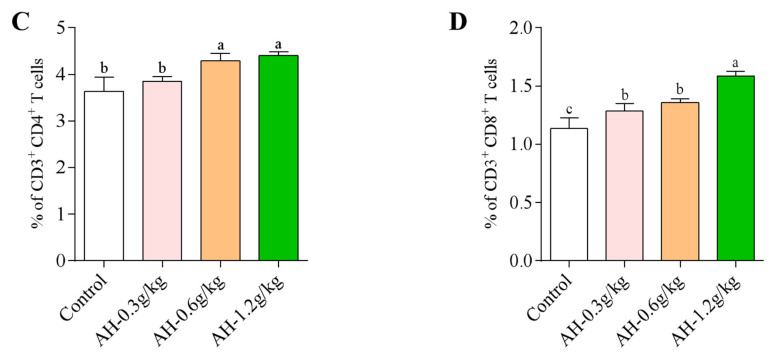
Effects of AH on the proliferation of peripheral blood immune cells after the experiment. (**A**,**B**) The representative flow cytometry plots of CD4^+^ and CD8^+^ T cells. Proportions of T-cell subpopulations CD4^+^ (**C**), and CD8^+^ (**D**) T cells in peripheral blood. Data were given as mean ± SEM. (n = 5). Bars in the figure without the same superscripts (a–c) differ significantly (*p* < 0.05).

To further investigate the effect of AH on the immune function in chicken, the ratio of CD8^+^ and CD4^+^ T cells in spleen and peripheral blood was analyzed using immunofluorescence and flow cytometry. As shown in Figure 5A–D, the AH-1.2 g/kg and AH-0.6 g/kg groups prominently enhanced the percentage of CD4^+^ T cells in the peripheral blood when comparison with control (*p* = 0.001, *p* = 0.002) and AH-0.3 g/kg groups (*p* = 0.006, *p* = 0.017), and compared to control group, the ratio of CD8^+^ T cells was remarkably enhanced after AH-treatment (0.3 g/kg, 0.6 g/kg, 1.2 g/kg) (*p* = 0.019, *p* = 0.002, *p* = 0.001). Moreover, the ratio of CD8^+^ T cells in AH-1.2 g/kg group was significantly higher than that in AH-0.3 g/kg (*p* = 0.001) and AH-0.6 g/kg groups (*p* = 0.002).

**Figure 6:**
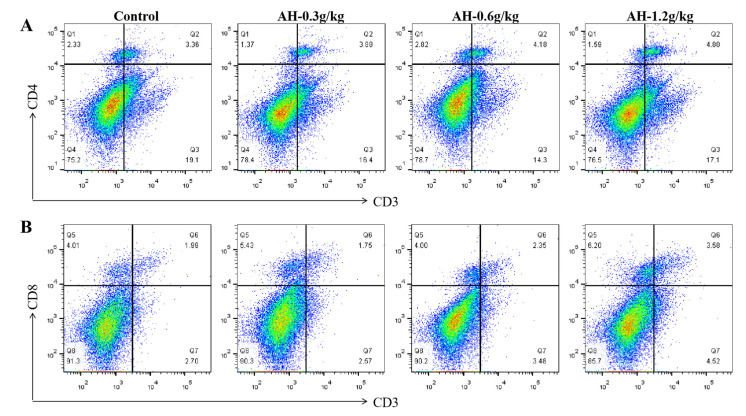

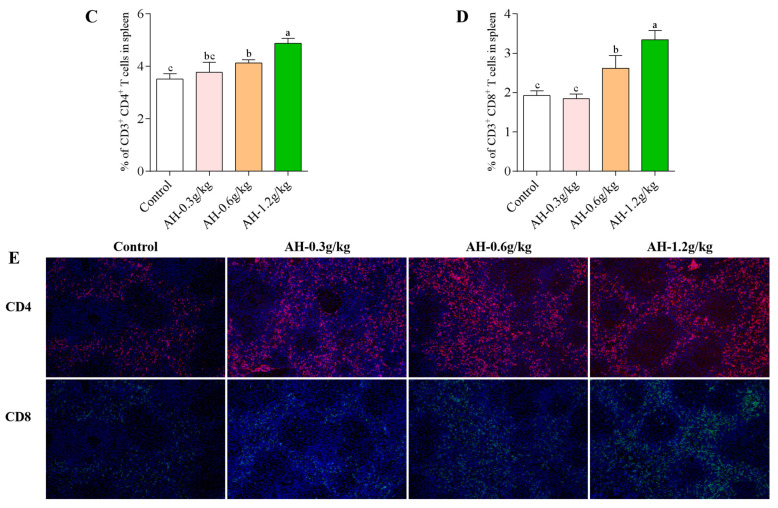
Effects of AH on the proliferation of spleen immune cells after the experiment. (**A**,**B**) The representative flow cytometry plots of CD4^+^ and CD8^+^ T cells. (**C**,**D**) Proportions of T-cell subpopulations CD4^+^ and CD8^+^ T cells in spleen. (**E**) Immunofluorescence of staining CD4 (red) or CD8 (green) and DAPI (blue) in spleen sections. Bars = 50 μm, magnification, 200×. Data were given as mean ± SEM. (n = 5). Bars in the figure without the same superscripts (a–c) differ significantly (*p* < 0.05).

Additionally, the proportion of CD4^+^ and CD8^+^ T cells in spleen was presented in Figure 6A–D. The AH-1.2 g/kg and AH-0.6 g/kg groups could significantly enhance the percentage of CD8^+^ (*p* = 0.001, *p* = 0.004) and CD4^+^ (*p* = 0.001, *p* = 0.016) T cells as compared to control group, and the percentage of CD8^+^ T cells in AH-0.3 g/kg group was remarkable lower than that in AH-1.2 g/kg (*p* = 0.001) and AH-0.6 g/kg (*p* = 0.002) groups. Moreover, the proportion of CD4^+^ and CD8^+^ T cells could be significantly enhanced by AH-1.2 g/kg group as compared the AH-0.6 g/kg group (*p* = 0.006, *p* = 0.003). Furthermore, immunofluorescence analysis of spleen slices found that the percentage of CD8^+^ and CD4^+^ T cells in the spleen was upregulated after AH-treatment, which was consistent with the flow cytometry results (Figure 6E).

## 3. Discussion

Modern poultry farming pays great attention to the health of the chicken, which directly affects the growth performance of the chicken. Currently, a lot of plant polysaccharide was used to maintain the health of chicken through improving the intestinal function and immunity [1,13]. In this study, the AH was used to improve the chicken’s intestinal function, gut microbiome, and immunity. We found that AH-1.2 g/kg could significantly promote the chicken’s growth performance, which was consistent with previous report that polysaccharides could improve chicken’s growth performance [6]. Moreover, the ratio of V/C was important indicators reflecting body’s digestion and absorption function [14]. The concentrations of SCFAs were the main fermenting product of dietary polysaccharides by gut microbiome, which were the important nutrient for intestinal absorption [15]. V/C and SCFAs were closely related to the poultry’s growth performance, and they have a positive correlation [14,16]. Our data suggested that AH could obviously enhance the ratio of V/C and the concentrations of SCFAs in intestine. These data demonstrated that AH could promote the chicken’s growth performance through increasing the percentage of V/C and the concentrations of SCFAs.

Additionally, intestinal flora was strongly associated with polysaccharide metabolism, intestinal health, and chicken’s growth performance [16]. In this experiment, we found that compared to the control group, the richness and diversity of intestinal microbiota in chicken were obviously enhanced after AH-treatment. The intestinal flora plays a vital role in protecting the physiological and immune functions of the gut, and maintaining gut health, and enhancing the growth performance of chicken [2,5]. An increase in the richness and diversity of intestinal microbiota can enhance intestinal immune function [3], otherwise it will increase the risk of intestinal infection [17,18]. Thus, our data have demonstrated that AH could enhance the richness and diversity of intestinal microbiota in chicken, thereby strengthening infection resistance to maintain gut health. Besides, at the phylum level, we found that the abundances of Bacteroidetes in AH-treatment group were significantly higher than that in control group, but the abundances of Proteobacteria in AH-treatment group were remarkably lower than that in control group. Bacteroidetes play an important role in degradation and absorption of polysaccharides and prefer to scavenge complex carbohydrates [15,19]. SCFAs as the primary product of polysaccharide fermentation have been significantly enhanced in the gut. These data suggested that AH could improve the microbiota structure to promote chicken’s growth performance. Furthermore, Bacteroidetes was also closely correlation with the balance of intestinal microflora and gut immunity [20]. Proteobacteria contained pathogens including *Salmonella*, *Helicobacter*, and *Vibrio spp*, which was greatly related to the gut disease [21]. Therefore, these data supported that the improvement of the gut immunity in chicken might be due to the ratio of beneficial bacteria increases and the percentage of intestinal pathogenic bacteria decreases at the same time.

Intestinal immune system was the vital defense barrier to maintain gut health. Secretory IgA (sIgA), as an important protective antibody in the intestinal mucosa, serves as a chemical barrier to protect the intestine from pathogen invasion [22]. Our data showed that AH could observably improve the sIgA secretion to strengthen intestinal immune function. Moreover, intraepithelial lymphocytes (IELs), as the vital T-cell populations in intestine, could keep the integrity of the intestinal epithelium through exploiting its effective immune response [23]. We also found that after AH-treatment, the number of IELs in intestine was significantly increased. sIgA and IELs are the important elements of intestinal immune system used to protect gut health. Thus, these results suggested that AH could improve the content of sIgA and IELs to maintain intestine health.

In addition, cytokines and CD4^+^ T cell are the important regulators of the gut immune system. IL-2 and INF-γ, as Th1 cells, could induce CD8^+^ cytotoxic T cells response [24]. IL-4 is secreted by Th2 cells and can further stimulate B cells to secrete antibodies [25]. IL-17 is responsible for eliminating pathogens which on the intestinal mucosa [26]. Our study showed that compared to control group, the content of cytokines in AH-treatment group was remarkably upregulated. Moreover, we also found that AH could significantly increase the ratio of CD8^+^ and CD4^+^ T cells in intestine as compared to the control group. CD4^+^ T cells could categorize into Th1 and Th2 cells to secrete different cytokines [26], and the invading pathogens in cells could be directly eliminated by CD8^+^ cytotoxic T cells [25]. These data suggested that AH could enhance the percentage of CD8^+^ and CD4^+^ T cells to increase the secretion of cytokines, thereby regulating the intestine immune system to maintain the gut health.

A healthy intestinal barrier is an important guarantee for exerting the physiological and immune functions of the intestine [27]. The intercellular tight junction (TJ) protein and the mucus layer are closely related to the functional integrity of intestinal mucosal epithelial cells [4,28]. The mucus layer that are secreted by the goblet cell are overlying intestinal epithelium and as the chemical barrier to prevent toxic molecules, parasite, and pathogens from invading the intestinal mucosa, thus protecting intestine function [29]. In this research, our data showed that AH-treatment group could remarkably enhance the number of goblet cells. Moreover, we also found that AH could significantly enhance the expression of Claudin-1, Occludin, and ZO-1 proteins as compared to control group. TJ (ZO-1, Occluding, and Claudin-1) proteins, as intercellular junctional complexes, are essential for maintaining the intestinal epithelial barrier function [30,31]. Thus, these data demonstrated that AH could strengthen the mucus layer and intercellular tight junctions to improve the intestinal epithelial barrier, thereby maintaining the health of intestine in chicken.

Immune function is a vital defense barrier for chickens to maintain healthy and efficient production. Spleen, thymus, and BF are the crucial immune organ of chickens, and the index of them is an important indicator to measure the immune function of chickens [32]. Peripheral blood and spleen lymphocytes, as a vital ingredient of organism immunity, are closely related to the humoral immunity and cellular immune response [25]. The CD4^+^ T cells can differentiate into Th1 and Th2 cells to regulate the humoral immunity and cellular immune response [33,34], and the CD8^+^ T cells could differentiate into CD8^+^ cytotoxic T cells, which could directly eliminate intracellular pathogen infection [25]. Our data suggested that AH could significantly enhance the ratio of CD8^+^ and CD4^+^ T cells in spleen and peripheral blood and the index of Spleen, thymus, and BF, suggesting the humoral and cellular immunity were strengthened [25]. These data proved that AH could maintain the health of chicken through improving the immune function of the chicken.

## 4. Materials and Methods

### 4.1. Materials

Alhagi honey was obtained from Xinjiang Madison Uygur Medicine Co., Ltd. Pieces Factory, Urumqi, China.

### 4.2. Preparation of Alhagi Honey Polysaccharides

AH was extracted as previously described [9]. In brief, Alhagi honey was decolorized and degreased by petroleum ether (60 °C, 2 h) and 95% ethanol (80 °C, 2 h). Then, Alhagi honey was extracted by water twice (80 °C, 2 h), the extract liquid was centrifuged (8000 rmp/min, 10 min), and then the 95% alcohol was added (alcohol:extract liquid/1:4). After standing for 24 h, the sample was centrifuged (8000 rmp/min, 10 min) and the precipitation was collected and redissolved in water. Then, the sample was further purified by using DEAE Sepharose Fast Flow and Sephadex G-100 (Beijing Solarbio Technology Co., Ltd., Beijing, China). Finally, AH which contained 99.0% carbohydrate was obtained by freeze-drying.

### 4.3. Animal Experimental Design

Chicken (Hy-Line Brown/male, one-day-old, 120) were bought from Nanjing Te Gei Li Poultry farm (Nanjing, China) and housed in wire cages. At the initial stage (0–3), the room temperature was kept about 32 °C, and then decreased by 0.5 °C every day until 24 °C at the rest of the experiment (during the whole experiment period, daily light was 12 h). After 7 days of acclimatization, all chicken were stochastically allocated into four groups (each group with 5 cages, 6 chicken per cage): Control group, AH-0.3 g/kg group, AH-0.6 g/kg group, and AH-1.2 g/kg group. At 9:00 am every day, all chickens were given oral intragastric administration of 0.5 mL different dosages (0.3 g/kg, 0.6 g/k, and 1.2 g/kg) AH with a 1 mL syringe, while the control group was given oral intragastric administration of 0.5 mL water for 14 days. All chicken were fed same diet and could freely drink water throughout the experiment. The main ingredients of the feed are listed in Table 2. The feed consumption of chicken was recorded daily, the body weight of chicken was recorded every 7 days. After the experiment, chicken were euthanatized with CO_2_.

### 4.4. Measurement of Growth Performance and the Index of Spleen, Thymus, and Bursa of Fabricius

During the experimental period, the feed consumption and body weight of chicken were recorded every day. Spleen, thymus, and bursa of fabricius (BF) of chicken (n = 8) were obtained and then their weights were recorded after the experiment. The index of spleen, thymus, and BF was calculated by the formula:

Index (g/kg) = immune organs weight/body weight

### 4.5. Determination of sIgA in Duodenal Washing Liquor

After the experiment, 8 cm sections of the duodenum (n = 8) were isolated and used to obtain the washed washing liquor [3]. Then, the mouse sIgA enzyme-linked immunosorbent assay (ELISA) kit (Wuhan Huamei Biotech Co., Ltd., Wuhan, China) was used to detect the level of sIgA in duodenal.

### 4.6. Detection of Cytokines in Duodenum

Duodenum samples (200 mg) (n = 8) were collected and homogenized to obtain the supernatant [3], which was used for testing the level of IL-17, IL-4, IL-2 and INF-γ by ELISA kits (Wuhan Huamei Biotech Co., Ltd.) under the instructions of the manufacturer.

### 4.7. CD4, CD8 Immunohistochemistry

The ileum (1cm, n = 5) and spleen of chicken (n = 5) were collected after the experiment. Ileums and spleens were made into paraffin sections (5 µm). Next, slides were deparaffinized before antigen retrieval and then blocked with goat serum (1:19 diluted in PBS). Then the presence of CD8^+^ and CD4^+^ T cells abundance in slides was detected using primary antibodies against CD4^+^ and CD8^+^, followed by incubation with secondary antibodies (CD4 was marked by CY3-red, CD8 was marked by FITC-green) 1 h at room temperature. Slides were evaluated using the fluorescence microscopy in a LEICA SP5 (Leica Microsystems, Wetzlar GmbH, Germany), CD4 and CD8 positive areas percentage was measured using three representative fields of view per section.

### 4.8. Intestinal Morphology

Intestinal morphology was analyzed through counting the number of IELs and measuring villus length and crypt depth. Chicken in each group were euthanatized after the experiment. Duodenum, jejunum, and ileum (1 cm) was separated from chicken (n = 5) and then fixed with 4% formalin. The samples were made into hematoxylin–eosin (H-E) slices according to standard protocols [3]. To analyze the intestinal morphology, sections were analyzed under a digital camera (Nikon, Japan). IELs were counted by analysis of 100 columnar epithelial cells from 5 gut villi in *per* section. Measurements were performed in duodenal, jejunum, and ileum sections by random measurement of 10 villi height (V)/crypts depth (C) per section.

### 4.9. Determination of the Number of Goblet Cells in Ileum

One centimeter of ileum in poultries (n = 5) was collected and fixed with 4% formalin. Then samples were made to 5 µm paraffin slices and then stained with periodic acid-Schiff (PAS). To count the number of goblet cells, sections were analyzed under an ordinary optical microscope. Count was performed in ileum by random counting of 5 intestinal villi in each section.

### 4.10. Quantitative PCR for Proteins of Claudin-1, Occludin, and ZO-1 in Ileum

Ileum samples (n = 8) were collected to extract RNA. Total RNA of ileum tissue was extracted using TRIzol reagent (Ambion by life technologies) [3,35]. In brief, the samples were homogenized in TRIzol reagent and stood for 10 min, then chloroform was added and shaken, then the supernatant was obtained by centrifugation (12,000 rmp/min, 10 min), isopropyl alcohol was used to precipitate the RNA in the supernatant, then the RNA was collected by centrifugation (12,000 rmp/min, 10 min). The RNA was washed by 75% alcohol for 3 times and then re-dissolved by diethylpyrocarbonate (DEPC) water (Vazyme Biotech Co., Ltd.). The reverse transcription Kit and quantitative PCR (qPCR) Mix Kit (Vazyme Biotech Co., Ltd.) were used to perform the reverse transcription and qPCR [3,36]. In brief, the RNA was detected with a spectrophotometer (NanoDrop ND-1000; Thermo Fisher Scientific, Wilmington, DE, USA). Then, the RNA was converted into cDNA by reverse transcription Kit. The cDNA was further used in the qPCR analyses. The qPCR reaction system was 20 µL, the 2×ChamQ Universal SYBR qPCR Master Mix was added 10 µL, the forward and reverse specific primers (10 µM) was 0.4 µL, respectively. The 2 µL cDNA and 7.2 µL ddH_2_O was added. The detail qPCR program was showed that the first step was pre degeneration (95 °C, 30 s), the second step was cyclic response (cycle: 40, 95 °C, 10 s, 60 °C, 30 s), the third step was to verify the specificity of primers (95 °C, 15 s, 60 °C, 60 s, 95 °C, 15 s). The primers of Claudin-1, Occludin, ZO-1 and GAPDH [16] were used and listed in supplementary Table S1. Relative gene expression was shown using the 2^−ΔΔCt^ method.

### 4.11. Measurement of SCFAs Concentrations in the Contents of Cecal

The samples (n = 8, 0.5 g cecal stool) were collected from the cecum in chicken. The SCFAs concentrations in the contents of cecal in poultry were analyzed by Gas chromatograph (GC) (Thermo Fisher Scientific, Waltham, MA, USA) [4].

### 4.12. Extraction of Fecal DNA and 16S rDNA Sequencing Analysis

The content of cecum was obtained after the experiment (n = 8). The samples of control group (ND1) and AH-1.2 g/kg group (AH1) were used for 16S rDNA sequencing analysis [3]. Briefly, the total genomic DNA was extracted from the sample of cecum using a E.Z.N.A.™ Bacterial DNA kit (Omega Bio-tek, Inc., Norcross, GA, USA). For 16S rDNA sequencing analysis, PCR amplifications with the universal primers 341F (5′-CCTACGGGNGGCWGCAG-3′) and 805R (5′-GACTACHVGGGTATCTAATCC-3′). Then, amplicons were tested for integrity and purified. Subsequently, a MiSeq Reagent Kit v3 (Illumina, San Diego, CA, USA) was used to construct the sequencing library. Then, the Illumina MiSeq Benchtop Sequencer (Illumina, San Diego, CA, USA) was used to sequence. Finally, the previous bioinformatics analysis method was used for sequence analysis [3].

### 4.13. The Ratio of CD4^+^ and CD8^+^ T Cells in Peripheral Blood and Spleen

The spleen and peripheral blood lymphocytes were obtained after the experiment (n = 5). The FITC-CD3, PE-CD4 and APC-CD8 antibodies (Southern Biotech) were used to stain the lymphocytes (peripheral blood and spleen) (30 min, 4 °C, avoid light). Next, lymphocytes were resuspended twice by PBS and centrifuged (4000 rpm, 4 °C, 5 min), and then resuspension in PBS. Finally, the ratio of CD8^+^ and CD4^+^ T cells were detected using the flow cytometer (BD Accuri C6).

### 4.14. Statistical Analysis

The data in the figures and tables were presented as mean ± SEM. Experimental data have been analyzed by the Shapiro-Wilk with SPSS v.21 (SPSS Inc., Chicago, IL, USA). The result showed that all the data conform to normal distribution, so the data were analyzed with one-way ANOVA and Tukey’s test used to analyze differences between groups with SPSS v.21. A probability value *p* < 0.05 was considered as statistically significant.

## 5. Conclusions

In summary, these results proved that the beneficial effect of AH on the growth performance, intestine function, and body immunity in chicken, which was evidenced by that AH enhanced the ADG while decreased the F:G, strengthened the intestinal chemical and mechanical barriers and nutrient absorption, and promoted the production of SCFAs and body immunity. In addition, AH can improve chicken’s intestinal flora structure by inhibiting pathogenic bacteria and increasing bacterial diversity to strengthen the intestinal immune system. Therefore, based on these, AH could be considered as a potential tool to maintain the chicken’s health and improve the growth performance.

## Figures and Tables

**Table 1:** Effect of Alhagi honey polysaccharides on the average daily gain, the average daily feed intake, and feed-to-gain ratio of chicken.

Items	Control	AH-0.3 g/kg	AH-0.6 g/kg	AH-1.2 g/kg	SEM	*p*-Value
D1 to D7 after first AH-treatment						
ADG, g/d	8.87	8.59	9.51	9.76	0.4	0.871
ADFI, g/d	21.31	21.23	22.28	22.28	0.17	0.083
F:G ratio	2.42	2.49	2.45	2.43	0.1	0.995
D8 to D14 after first AH-treatment						
ADG, g/d	12.01 ^b^	12.87 ^ab^	13.65 ^ab^	14.5 ^a^	0.33	**0.024**
ADFI, g/d	26.41	25.26	26.11	25.72	0.17	0.082
F:G ratio	2.2 ^a^	1.97 ^ab^	1.95 ^ab^	1.78 ^b^	0.05	**0.011**

Means within a row without the same superscripts (a,b) differ significantly (*p* < 0.05). ADG, average daily gain; ADFI, average daily feed intake; F:G, feed-to-gain ratio; AH, Alhagi honey polysaccharides (n = 5).

**Table 2:** Composition of the diet in the experiment.

Ingredients (%)		Chemical Composition Calculated (%)	
Corn	54.69	Metabolizable energy (MJ/kg)	2.89
Soybean meal	32.79	Crude protein	22.47
Fish meal	3.09	Calcium	1.05
Soybean oil	2.36	Available phosphorus	0.55
Limestone	1.03	Lysine	1.23
Bicalcium phosphate	1.89	Methionine	0.54
Salt	0.35	Methionine + cystine	0.85
DL-Methionine	1.75		
L-Lysine	0.84		
Vitamin-mineral premix ^a^	1.2		
Total	100		

^a^ Provided per kilogram of diet: 5 g of vitamin E, 150,000 IU of vitamin D3, 1, 200, 000 IU of vitamin A, 1.8 g of vitamin K3, 0.5 g of vitamin B1, 2.0 g of vitamin B2, 0.1 g of vitamin B6, 20 mg of vitamin B12, 1.5 g of nicotinic acid, 0.5 g of pantothenic acid, 20 mg of folic acid, 20 mg of biotin, 15 mg of Cu, 75 mg of Fe, 90 mg of Zn, 110 mg of Mn, 0.5 mg of Se, 0.5 mg of I.

## Data Availability

The data presented in this study are available on request from the corresponding author.

## Supplementary Materials

The following supporting information can be downloaded at: https://www.mdpi.com/article/10.3390/ijms232214332/s1.
