# Supplementary material for: Effects of Alhagi Honey Polysaccharides as Feed Supplement on Intestine Function and Microbiome, Immune Function, and Growth Performance in Chicken
Source: Int J Mol Sci. 2022 Nov 18;23(22):14332. doi: 10.3390/ijms232214332 (PMC9694483; doi:10.3390/ijms232214332)
Supplement: Supplementary file 1 [file ijms-23-14332-s001.zip › ijms-2023156-supplementary.pdf]

Effects of Alhagi honey polysaccharides as feed supplement on intestine function and microbiome, immune function, and growth performance in chicks

SUPPLEMENTAL INFORMATION

Table S1. The primer list of *Claudin-1*, *Occludin*, *ZO-1*, and *GAPDH* protein.

Gene	Primer sequence (5’-3’)
Claudin-1	F: TGATTGCTTCCAACCAGGCT<br>R: CACACGGCTCTCCTTGTCTA
Occludin	F: ATCGCCTCCATCGTCTACATC<br>R: GCTGCACATGGCCAACAAG
ZO-1	F: TGGGCCTCACGGACTAAAAT<br>R: GTTTGCTCCAACAAGATAGTTTGG
GAPDH	F: GGAAAGTCATCCCTGAGCTGAAT<br>R: GGCAGGTCAGGTCAACAACA
